# Supplementary material for: Whole-Genome and Poly(A)+Transcriptome Analysis of the Drosophila Mutant agnts3 with Cognitive Dysfunctions
Source: Int J Mol Sci. 2024 Sep 13;25(18):9891. doi: 10.3390/ijms25189891 (PMC11432035; doi:10.3390/ijms25189891)
Supplement: Supplementary file 1 [file ijms-25-09891-s001.zip › Supplementary materials/Table S1.pdf]

Strains	DEGs ↑ (genes)	DEGs ↓ (genes)	DEGs ↑ (isoforms)	DEGs ↓ (isoforms)	Enrichment: DEGs (KEGG)	Enrichment: DEGs (GO Biological Processes)	Network (GO Biological Process)
agn^{ts3} vs *CS*	*CR34335, PGRP-SC1b, Copia{}997, flea{}102, CG30428* (138)	*sordd2, gypsy10{}6526, prosalpha1, TotM, TotC* (155)	*porin-RA, Npl4-RD, CG5162-RD, CNX99A-RB, CG16826-RB* (273)	*TotM-RB, CG10550-RB, Lectin-galC1-RA, Actn-RH, sordd2-RA* (340)	↑ Toll and Imd S.;<br>↓ Glycosaminoglycan B. -chondroitin sulfate/dermatan sulfate, galactose M., glycolysis/ gluconeogenesis, lysosome	↑ Peptidoglycan M./C., glycosaminoglycane C., defense response to Gram-positive bacterium,<br>↓ Cellular response to UV, cellular response to heat, response to bacterium	↓ Proteolysis, cellular response to stress, response to temperature stimulus, response to heat, cellular response to heat
agn^{ts3} (HMC) vs *CS* (HMC)	*PGRP-SC1b, CR34335, CG30428, CR44344, CR43084* (134)	*sordd2, gypsy10{}6526, flea{}877, TotM, SPH93* (160)	*Ald1-RD, Pgant1-RB, apolpp-RD, Act5C-RA, porin-RA* (303)	*CG10550-RB, lncRNA:ROX1-RD, Act5C-RC, Actn-RH CG8708-RC,* (374)	↑ Toll and Imd S;<br>↓ Starch and sucrose M., galactose M., pyruvate metabolism, glycolysis/ gluconeogenesis	↑ Negative regulation of B. of antibacterial peptides active against Gram-negative bacteria, negative regulation of antibacterial peptide production and B.<br>↓ Cellular response to heat, response to bacterium, defense response to other organisms	No
CS (HMC) vs *CS*<br><br>[no Wald test]	No [*HMS-Beagle{}101 HMS-Beagle{}1014* (2)]	No [*Rover{}6572, MtnD, CG12057, phu, MtnC, iotaTry* (7)]	*tweek-RG, unc-13-RB, Pgant1-RA, simj-RA, Vap33-RC* (26)	*Pgant1-RA, CG12376-RB, Lectin-37DA-RA, Khc-73-RA, gel-RJ* (14)	No<br><br>[↓ Folate B., Neuroactive ligand-receptor interaction]	No<br><br>[↓ Response to nicotine, response to copper ion, response to metal ion.]	No<br><br>[No]
agn^{ts3} (HMC) vs agn^{ts3}	*prosalpha1* (1)	No	*Bbx-RD, Hsc70Cb-RF, prosalpha1-RB, aru-RA, CG31064-RF* (27)	*CG9279-RC, CG7029-RC, CG31821-RB, CDA5-RA, CG6907-RB* (25)	No	No	No
agn^{ts3} (HS) vs *CS* (HS)	*flea{}1074, Dm88{}1662, CR42722, CR44344, PGRP-SC1b* (97)	*CR40469, CG31205, CG44102 Mdg3{}291, CG44535* (113)	*porin-RA, CG5162-RD, Actn-RG, CG16826-RB, lncRNA:ROX1-RC* (194)	*futsch-RC, Plp-RK, dnt-RB, Dhc64C-RE, CG10550-RB* (204)	↑ Hippo S. - multiple species, homologous recombination, apoptosis – multiple species, fanconi anemia pathway, Toll and Imd S.	↑ Negative regulation of B. of antibacterial peptides active against Gram-negative bacteria / Negative regulation of antibacterial peptide production	No
CS (HS)	*Hsp70Aa,*	*CG42305,*	*futsch-RC,*	*Culd-RA,*	↑ Caffeine M.,	↑ Heat shock-mediated polytene	↑ Chromosome organization,

vs *CS*	*Hsp70Bb, Hsp70bbb, Hsp70Ab, Hsp70Bc* (66)	*CG17325, CG34426* (3)	*E(BX)-RE, DHC64C-RE, KDM5-RA, CG42671-RK* (116)	*CG5011-RA, CG1677-RC, CG8026-RC, wnd-RA* (37)	longetivity regulating pathway – multiple species, protein processing in EPR, spliceosome, endocytosis	chromosome puffing , protein refolding, chaperone-mediated protein folding	cellular response to organic substance, response to unfolded protein, cellular response to heat, de novo protein folding
agn^{ts3} (HS) vs *agn*^{ts3}	*Hsp70Aa, Dm88{}1691, Dm88{}1694, CR32865, Hsp70 Ab* (77)	*3S18{}5536, CG42305, CG17325, CG34426* (4)	*cpx-RS, CG32809-RL, HSP70AA-RA DM88{}1691, DM88{}1694* (154)	*Culd-RA, CG8026-RC, msps-RB, Pld-RD, CycG-RA* (75)	↑ Caffeine M., other glycal degradation, longetivity regulating pathway – multiple species, protein processing in EPR, spliceosome, endocytosis	↑ Heat shock-mediated polytene chromosome puffing , protein refolding, chaperone-mediated protein folding	↑ Chromosome organization, cellular response to organic substance, response to unfolded protein, cellular response to heat, de novo protein folding

Table S1. Differentially expressed genes in *CS* and *agn*^{ts3} with the highest values of expression change.

Abbreviations. ↑ – upregulated, ↓ – downregulated, B. – biosynthesis, EPR – endoplasmic reticulum, M. – metabolism, S. – signaling pathways, UV - ultraviolet. Up to 5 DEGs with increased and decreased expression are shown. In brackets – the total number of DEGs. Genes and the main enriched processes are shown in descending order of fold enriched.
