# Supplementary material for: Whole-Genome and Poly(A)+Transcriptome Analysis of the Drosophila Mutant agnts3 with Cognitive Dysfunctions
Source: Int J Mol Sci. 2024 Sep 13;25(18):9891. doi: 10.3390/ijms25189891 (PMC11432035; doi:10.3390/ijms25189891)
Supplement: Supplementary file 1 [file ijms-25-09891-s001.zip › Supplementary materials/Text S1.pdf]

Text S1. The procedure of assembling and analysis of genomes and transcriptomes

a. DNA sequences assembly and annotation

I. Using Trimmomatic to trim bad ends of reads and remove unpaired reads for the first (1) and second (2) sequences parties:

For *CS* (1) DNA sequences (forward and reverse: Cs-DNA_1.fq.gz, Cs-DNA_2.fq.gz):
java -jar trimmomatic-0.39.jar PE -threads 6 -phred33 Cs-DNA_1.fq.gz Cs-DNA_2.fq.gz CS-tr-paired_1.fastq.gz CS-tr-unpaired_1.fastq.gz CS-tr-paired_2.fastq.gz CS-tr-unpaired_2.fastq.gz LEADING:15 TRAILING:15 SLIDINGWINDOW:4:22 MINLEN:36

For *CS* (2):
java -jar trimmomatic-0.39.jar PE -threads 6 -phred33 CS-DNA-2_1.fq.gz CS-DNA-2_2.fq.gz CS2-tr-paired_1.fastq.gz CS2-tr-unpaired_1.fastq.gz CS2-tr-paired_2.fastq.gz CS2-tr-unpaired_2.fastq.gz LEADING:15 TRAILING:15 SLIDINGWINDOW:4:22 MINLEN:36

For agn^{ts3} (1):
java -jar trimmomatic-0.39.jar PE -threads 6 -phred33 Agn-DNA_1.fq.gz Agn-DNA_2.fq.gz Agn-tr-paired_1.fastq.gz Agn-tr-unpaired_1.fastq.gz Agn-tr-paired_2.fastq.gz Agn-tr-unpaired_2.fastq.gz LEADING:15 TRAILING:15 SLIDINGWINDOW:4:22 MINLEN:36

For agn^{ts3} (2):
java -jar trimmomatic-0.39.jar PE -threads 6 -phred33 Agn-DNA-2_1.fq.gz Agn-DNA-2_2.fq.gz Agn2-tr-paired_1.fastq.gz Agn2-tr-unpaired_1.fastq.gz Agn2-tr-paired_2.fastq.gz Agn2-tr-unpaired_2.fastq.gz LEADING:15 TRAILING:15 SLIDINGWINDOW:4:22 MINLEN:36

II. Sequences assembly

1. Indexing of reference genome sequence (Drosophila_melanogaster.BDGP6.46.dna_sm.toplevel.fa, = Dm.fa):
bwa index Dm.fa

2. Assembly of *CS* and agn^{ts3} sequences:

For *CS* (1)
bwa mem Dm.fa CS-tr-paired_1.fastq.gz CS-tr-paired_2.fastq.gz > CS.sam
samtools view -bSq 32 CS.sam > CS.bam
samtools sort -O bam -o CS-sorted.bam -T temp CS.bam
samtools index CS-sorted.bam

For agn^{ts3} (1)
bwa mem Dm.fa Agn-tr-paired_1.fastq.gz Agn-tr-paired_2.fastq.gz > agn.sam
samtools view -bSq 32 agn.sam > agn.bam
samtools sort -O bam -o agn-sorted.bam -T temp agn.bam
samtools index agn-sorted.bam

For *CS* (2)

```
bwa mem Dm.fa CS2-tr-paired_1.fastq.gz CS2-tr-paired_2.fastq.gz > CS2.sam
samtools view -bSq 32 CS2.sam > CS2.bam
samtools sort -O bam -o CS2-sorted.bam -T temp CS2.bam
samtools index CS2-sorted.bam

# For agn^ts3 (2):
bwa mem Dm.fa Agn2-tr-paired_1.fastq.gz Agn2-tr-paired_2.fastq.gz > agn2.sam
samtools view -bSq 32 agn2.sam > agn2.bam
samtools sort -O bam -o agn2-sorted.bam -T temp agn2.bam
samtools index agn2-sorted.bam

# 3. Merging two sequences assemblies (for parties 1 and 2) to one:
samtools merge -X CS-full.bam CS-sorted.bam CS2-sorted.bam CS-sorted.bam.bai CS2-sorted.bam.bai
samtools merge -X agn-full.bam agn-sorted.bam agn2-sorted.bam agn-sorted.bam.bai agn2-sorted.bam.bai

#. III. Sequences annotation

# 1. VCF files creating:
bcftools mpileup -Ou -f Dm.fa CS-full.bam | bcftools call -mv -Oz -o CS.vcf.gz
bcftools mpileup -Ou -f Dm.fa agn-full.bam | bcftools call -mv -Oz -o agn.vcf.gz

# 2. Indels normalization:
bcftools index CS.vcf.gz
bcftools norm -f Dm.fa CS.vcf.gz -Ob -o CS-norm.bcf.gz
bcftools index agn.vcf.gz
bcftools norm -f Dm.fa agn.vcf.gz -Ob -o agn-norm.bcf.gz

# 3. Indels filtering:
bcftools filter --IndelGap 10 CS-norm.bcf.gz -Ob -o CS-final.bcf.gz
bcftools filter --IndelGap 10 agn-norm.bcf.gz -Ob -o agn-final.bcf.gz

# 4. Filtering SNPs of low quality and depth read:
bcftools view -i '%QUAL>=20 & DP >10' CS-final.vcf > CS-filtered-Q20-DP10.vcf
bcftools view -i '%QUAL>=20 & DP >10' agn-final.vcf > agn-filtered-Q20-DP10.vcf

# 5. Preparing the whole VCF files for annotation:
bgzip CS-filtered-Q20-DP10.vcf
bcftools index CS-filtered-Q20-DP10.vcf.gz
bgzip agn-filtered-Q20-DP10.vcf
bcftools index agn-filtered-Q20-DP10.vcf.gz

# 6. snpEff annotation of the whole VCF files:
java -jar snpEff.jar ann dmel_r6.12 CS-filtered-Q20-DP10.vcf.gz > CS-annotated.vcf
java -jar snpEff.jar ann dmel_r6.12 agn-filtered-Q20-DP10.vcf.gz > agn-annotated.vcf

# 7. Preparing VCF files with CS-specific and agn^ts3-specific changes:
bcftools isec CS-filtered-Q20-DP10.vcf.gz agn-filtered-Q20-DP10.vcf.gz -p isec
```

Folder isec contains VCF files with common, *CS*-specific and *agn^{ts3}*-specific changes. Files should be renamed manually according to their content.

8. Preparing the strain-specific VCF files for annotation:

CS-specific:
bgzip CS-specific.vcf
bcftools index CS-specific.vcf.gz

agn^{ts3}-specific:
bgzip agn-specific.vcf
bcftools index agn-specific.vcf.gz

Common for *CS* and *agn^{ts3}*:
bgzip Common.vcf
bcftools index Common.vcf.gz

9. snpEff annotation of the strain-specific and common VCF files:
java -jar snpEff.jar ann dmel_r6.12 CS-specific.vcf.gz > CS-specific-annotated.vcf
java -jar snpEff.jar ann dmel_r6.12 agn-specific.vcf.gz > agn-specific-annotated.vcf
java -jar snpEff.jar ann dmel_r6.12 Common.vcf.gz > Common-annotated.vcf

b. RNA sequences assembly and expected counts computation.

1. Genome indexation.
STAR --runThreadN 4 --runMode genomeGenerate --genomeDir Genome --genomeFastaFiles Dm.fa --sjdbGTFfile Dm.gtf --sjdbOverhang 100 --genomeSAindexNbases 12

Example: CS1N sequences (forward and reverse: CS1N_1.fq.gz, CS1N_2.fq.gz):

2. Transcriptome assembly
STAR --runThreadN 4 --genomeDir Genome --readFilesIn CS1N_1.fq.gz CS1N_2.fq.gz --sjdbGTFfile Dm.gtf --alignIntronMin 44 --alignIntronMax 70000 --readFilesCommand gunzip -c --outSAMtype BAM SortedByCoordinate --quantMode TranscriptomeSAM

3. Preparing transcript references
rsem-prepare-reference --transcript-to-gene-map gene_name_map.txt --polyA -gtf Dm.gtf Dm.fa Dm

4. Estimating genes and isoforms expression.
rsem-calculate-expression --alignments --paired-end --append-names Aligned.toTranscriptome.out.bam Dm CS1
