# Supplementary material for: Whole-Genome and Poly(A)+Transcriptome Analysis of the Drosophila Mutant agnts3 with Cognitive Dysfunctions
Source: Int J Mol Sci. 2024 Sep 13;25(18):9891. doi: 10.3390/ijms25189891 (PMC11432035; doi:10.3390/ijms25189891)
Supplement: Supplementary file 1 [file ijms-25-09891-s001.zip › Supplementary materials/Text S2.pdf]

Text S2. Primer sequences and PCR reaction parameters.

The following primer sequences were used for PCR:

rpl32
Forward: 5'-TATGCTAAGCTGTCGCACAAATGGC-3'
Reverse: 5'-GTTCTGCATGAGCAGGACCTCCA-3'

EF1α2
Forward: 5'-GCGTGGGTTTGTGATCAGTT-3'
Reverse: 5'-GATCTTCTCCTTGCCCATCC-3'

Cry #1 (PP16560)
Forward: 5'-AGGGTATAGCCCTAATTCCCG-3'
Reverse: 5'-GCATCCGATTGTAACCCACATT-3'

Cry #2, exon spanning primers (PP3400)
Forward: 5'-GGGCGAATGTGATTTGGTTTC-3'
Reverse: 5'-GGGAATTAGGGCTATACCCTGAT-3'

SPN42DE (PP22216)
Forward: 5'-CTTGGCCCTAGCCTACCTG-3'
Reverse: 5'-ACAACTGGTCCAATTTCTCGG-3'

HSP70Bbb (PD40148)
Forward: 5'-CGACGAGGGATCTCTGTTCG-3'
Reverse: 5'-GGTGACTAGCCGGTTGTCAA-3'

prosalpha1 (PD40683)
Forward: 5'-CCGGCTTTGACAGACACATC-3'
Reverse: 5'-ACTTGGTAGAGGCGTCCCT-3'

Numbers in brackets are FlyPrimerBank primer pair IDs (https://www.flyrnai.org/flyprimerbank)

PCR parameters
1. 1 cycle: 95 ºC— 5 min.
2. 50 cycles: 95ºC— 20 s; 58 ºC — 20 s; 72 ºC — 20 s; 75/ 76 /77 ºC *(Cry #1, EF1α2, prosalpha1 /Cry #2/ rpl32, SPN42DE, HSP70Bbb)* — 15 s (detection).
3. Melting curve: 95 ºC– 15 s, 60 ºC – 1 min, 60-95 ºC (Δ 0.3 ºC, 15 s).
